# Hyperglycemia and cancer in human lung carcinoma by means of Raman spectroscopy and imaging

**DOI:** 10.1038/s41598-022-21483-y

**Published:** 2022-11-03

**Authors:** M. Kopeć, K. Beton, K. Jarczewska, H. Abramczyk

**Affiliations:** Laboratory of Laser Molecular Spectroscopy, Institute of Applied Radiation Chemistry, Lodz University of Technology, Wroblewskiego 15, 93-590 Lodz, Poland

**Keywords:** Imaging studies, Raman spectroscopy, Cell biology, Cancer

## Abstract

Raman spectroscopy and Raman imaging were used to identify the biochemical and structural features of human cancer lung cells (CCL-185) and the cancer cells supplemented with glucose and deuterated glucose at normal and hyperglycemia conditions. We found that isotope substitution of glucose by deuterated glucose allows to separate de novo lipid synthesis from exogenous uptake of lipids obtained from the diet. We demonstrated that glucose is largely utilized for de novo lipid synthesis. Our results provide a direct evidence that high level of glucose decreases the metabolism via oxidative phosphorylation in mitochondria in cancer cells and shifts the metabolism to glycolysis via Warburg effect. It suggests that hyperglycemia is a factor that may contribute to a more malignant phenotype of cancer cells by inhibition of oxidative phosphorylation and apoptosis.

## Introduction

Sugars are molecules that contain carbon, hydrogen and oxygen atoms. They play an important role in the human body. First of all sugars are a source of energy to fuel human body^1^. Moreover sugars are one of the most important regulators of physiological processes in body such as growth, stress responses.

Diabetes is defined as a metabolic disease characterized by elevated levels of blood sugar, which leads to damage to many of the body’s organs. According to World Health Organization diabetes is called twenty-first century epidemic^2^. It is estimated that the number of obese will increase more rapidly in developing regions of the world^3^.

The values for normal fasting blood glucose concentration are between 70 and 100 mg/dL (3.9–5.6 mmol/L). Higher level of blood glucose is regarded as hyperglycemia.

Isotope substitution has been used to study metabolic processes and their molecular mechanisms of individual living cells^4–20^.

Various existing techniques of molecular biology methods such as enzyme-linked immunosorbent assays (ELISA), Western blot, high performance liquid chromatography (HPLC), spectrophotometry and flow cytometry can be used to estimate the concentration of metabolites. Isotope substitution is also very useful and has been extensively used in mass spectrometry (MS), nuclear magnetic resonance spectroscopy (NMR) and Raman spectroscopy to identify and quantify metabolites with high sensitivity^4,5,7–15^.

However, most of the methods (MS, NMR) provide only bulk analyses of cell populations and cannot monitor the biochemical heterogeneity within specific organelles of a single cell resulting in an inability to detect the full extent of metabolite localization inside and outside specific organelles. Moreover, none of the methods used to control metabolites concentration can provide direct evidence about their role in apoptosis and oxidative phosphorylation, because they are not able to monitor the amount of concentration in specific organelles such as mitochondria, cytoplasm, or extracellular matrix. Raman imaging is a new tool that can overcome these limitations. In Raman imaging we do not need to disrupt cells to release the cellular structures to learn about their biochemical composition^21–27^. Recently isotopic substitution has been used in linear and nonlinear regimes of Raman imaging^28–30^.

Increasing evidence suggests a close association between diabetes and cancer; however, the links between the two diseases remain still unclear^27,31,32^.

In this paper, we applied Raman imaging to study metabolism of human cancer lung cell line (CCL-185) and the cell line supplemented with glucose and deuterated glucose in normal and hyperglycemia conditions. We will concentrate on de novo lipogenesis, which is being increasingly recognized as a hallmark of cancer^33–35^.

The aim of this paper is to demonstrate the possibility of monitoring glucose metabolism occurring in cancer cells by Raman spectroscopy and Raman imaging. In this paper, we have analyzed the biochemical composition of specific organelles in lung cancer cells CCL-185 and the cancer lung cells supplemented with glucose and deuterated glucose.

Understanding sugar’s role in lung cancer using Raman spectroscopy will help establish a modern clinical diagnosis tools for diabetes and cancer monitoring.

## Materials and methods

### Chemicals

The nondeuterated glucose d-( +)-glucose (product number G7021) and deuterated glucose d_7_ d-glucose-1,2,3,4,5,6,6-d_7_ (product number 552003) were purchased from MERCK.

### Raman spectroscopy

All Raman spectra and Raman images presented in this manuscript were recorded using WITec alpha 300 RSA + combined with a confocal microscope coupled via the fibre of a 50 µm core diameter with an UHTS (Ultra High Throughput Spectrometer) spectrometer and a CCD Camera (Andor Newton DU970N-UVB-353) operating in standard mode with 1600 × 200 pixels at − 60 °C with full vertical binning. All experiments were performed using a laser diode (SHG of the Nd:YAG laser (532 nm)) and 40× Nikon water dipping objective (NA = 1.0). All cells were measured using laser with a power 10 mW at the sample position. The Raman images for all cells were recorded with integration time 0.3 s in the high frequency region and with 0.5 s in the fingerprint region. Acquisition and preprocessing of the data (cosmic rays removing, smoothing and removing background) were performed with WITec Project Plus software. The average Raman spectra were obtained for around 440 spectra for each organelle in a cell. The total number of cells was n = 23. The number of cells supplemented with glucose was n = 8. The number of cells supplemented with deuterated glucose was n = 7. The number of control cells was n = 8.

The Raman maps were created by Cluster Analysis method^36,37^.

Briefly, Cluster Analysis explore data analysis in which Raman spectra are divided into different groups that have some specific characteristic vibrational features. Cluster Analysis constructs separate groups (clusters). Each of the cluster (the Raman spectra) must be as similar as possible in contrast to the Raman spectra belonging to another groups. The separation of n observations (x) into k (k ≤ n) clusters S should be done to minimize the variance (Var) according to the formula:

$$\arg \;\min_{s} \sum\limits_{i = 1}^{k} {\sum\limits_{x \in Si} {\left\| {x\mu_{i} } \right\|}^{2} } = \arg \;\min_{s} \sum\limits_{i = 1}^{k} {\left| {S_{i} } \right|} VarS_{i}$$

where μ_i_ is the mean of experimental points. The Raman maps presented in the paper were constructed based on the principles of Cluster Analysis described above. The number of clusters was 7. Each cluster is characterized by specific average Raman spectra, which reflects the inhomogeneous distribution of chemical components within the organelles of single cells.

### Statistical analysis

The statistical analysis of the spectroscopic data was performed by using the one-way ANOVA test implemented in Origin software. The Tukey test was used to calculate the value of statistical significance, asterisk * denotes that the differences are statistically significant, p-value ≤ 0.05.

The normalization (model: divided by norm (divide the spectrum by the dataset norm)) were performed by using Origin software. The normalization model: divided by norm was performed according to the formula:

$${V}^{{\prime}}=\frac{V}{\parallel V\parallel }$$

$$\parallel V\parallel =\sqrt{{{v}_{1}^{2}+v}_{2}^{2}+\cdots {v}_{n}^{2}}$$

where: ${v}_{n}$ is the nth V value.

### Cell lines and cell culture

A549 cell line (ATCC^®^ CCL-185™) was purchased from ATCC: The Global Bioresource Center. CCL-185 cell line was cultured using ATCC-formulated F-12K Medium (Kaighn's Modification of Ham's F-12 Medium), Catalog No. 30-2004, contains 2 mM l-glutamine and 1500 mg/L sodium bicarbonate. Fetal Bovine Serum (FBS) was added to a medium to obtain a final concentration of 10%. The culture medium was renewed from 2 to 3 times a week. The CCL-185 cells were obtained from the lung tissue of a 58-year-old Caucasian male with lung cancer. The biological safety of the CCL-185 cell line has been classified by the American Biosafety Association (ABSA) as level 1 (BSL-1).

### Cultivation conditions

The cell line (CCL-185) used in the experiments in this study was grown in flat-bottom culture flasks made of polystyrene with a cell growth surface of 75 cm^2^. Flasks containing cells were stored in an incubator providing environmental conditions at 37 °C, 5% CO_2_, 95% air.

### Cell treatment with glucose and deuterated glucose

Cells used for research were seeded onto CaF_2_ windows (25 × 1 mm) at a low density of 104 cells/cm^2^. After 24 h incubation on the CaF_2_, standard growth medium was removed, and glucose or deuterated glucose in concentrations simulating normal (5 mM) and hyperglycemic (100 mM) conditions solution was added for 24 h. After 24 h, the cells were rinsed with Phosphate Buffered Saline (PBS, Gibco, 10010023, pH 7.4 at 25 °C, 0.01 M) to remove any residual medium and an excess of additives that did not penetrate inside the cells. Then, PBS was removed, and cells were fixed in Formaldehyde (4% buffered formalin) for 10 min and washed once more with PBS. The Raman confocal measurements were made immediately after the preparation of the samples. All the glucose solutions used for supplementation procedure in investigation were prepared by diluting in the pure culture medium dedicated to cell lines used in investigation as a solvent. For the preparation of glucose and deuterated glucose solutions, powdered reagents were used, from which samples were prepared to finally obtain a solution with concentrations of 5 and 100 mM.

## Results and discussion

**Figure 1:**
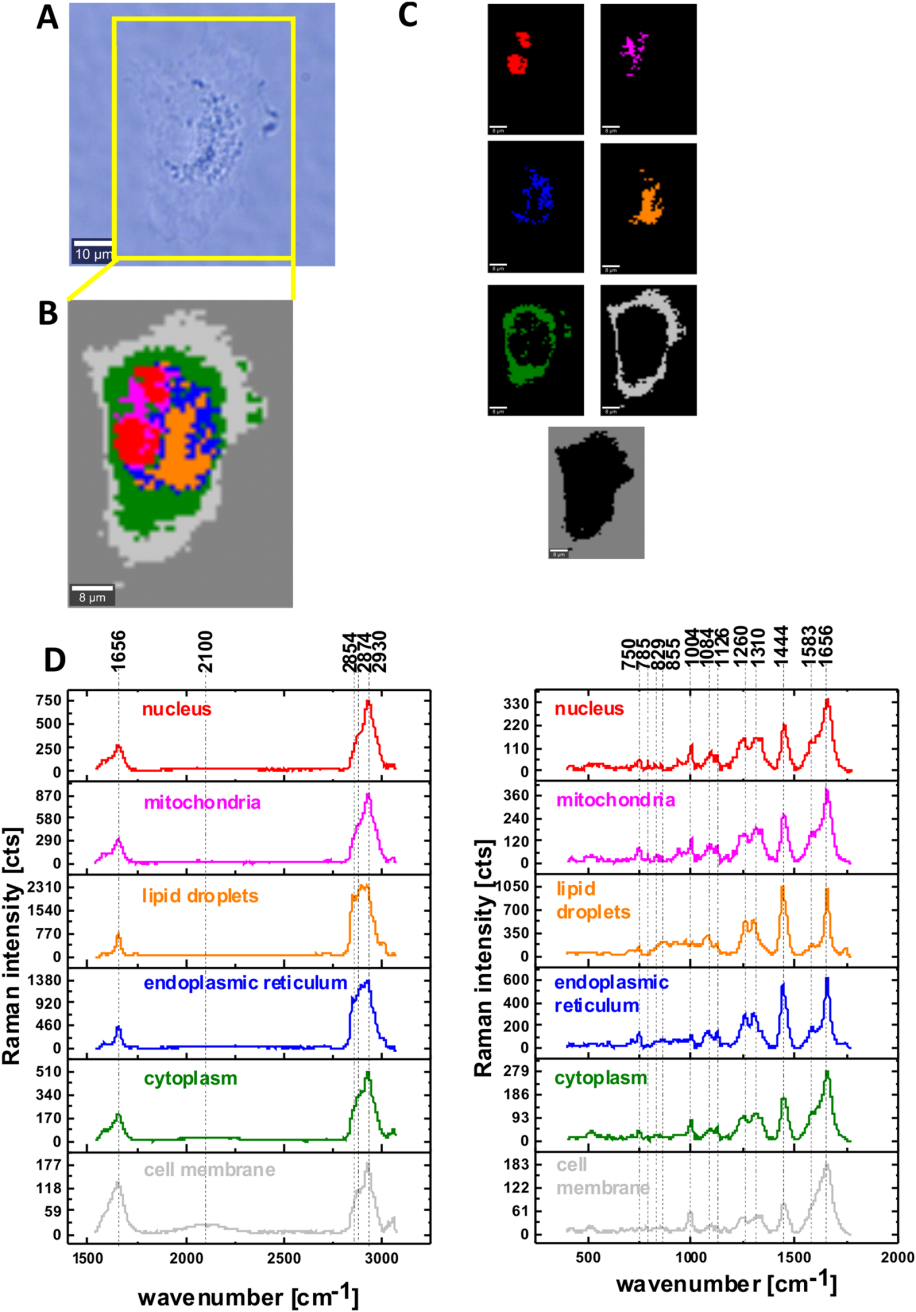
The microscopy image (**A**), Raman image for the area marked by yellow frame in the panel A, the size of Raman image (44 μm × 60 μm), resolution 1 μm of a typical human lung cell CCL-185 at normal glucose conditions 5 mM (**B**), Raman images of separate clusters identified by Cluster Analysis method assigned to: nucleus (red), mitochondria (magenta), endoplasmic reticulum (blue) and lipid droplets (orange), cytoplasm (green), cell membrane (light grey) and cell environment (dark grey) (**C**), the average Raman spectra for all clusters for high and for low frequency region (**D**), colors of the spectra correspond to the colors of clusters; integration time 0.3 s in the high frequency region and 0.5 s in the fingerprint region, laser power 10mW.

First, we focused on Raman imaging analysis for lung cancer cells CCL-185 at normal glucose conditions (5 mM). Figure 1 presents a typical video image, Raman images and Raman spectra for low and high frequency regions of CCL-185 human lung cancer cell.

To define the main biochemical components in specific organelles of cancer lung cells we used Cluster Analysis method to analyse the Raman spectra in the cells. In Fig. 1B shows the Raman image of a typical cell, panel C presents the clusters identified by the Cluster Analysis. The red area shows the locations of nucleus, magenta areas show the locations of mitochondria, orange and blue areas reflect the locations of lipids in lipid droplets and rough endoplasmic reticulum respectively, green areas reflect the locations of cytoplasm, light gray areas reflect the locations of cell membrane and dark gray areas reflect the locations of cell environment.

The results presented in Fig. 1D confirm that Raman imaging can characterize the biochemical composition of organelles in human lung cells. One can see that a typical cell in the fingerprint region is dominated by the peaks at: 750, 785, 829, 855, 1004, 1126, 1260, 1310, 1444, 1583, 1656 cm^−1^ and by the peaks at: 2854, 2874, 2930 cm^−1^ in the high frequency region. Table 1 presents the main chemical components which can be identified based on their Raman vibrational features.

**Table 1:** Band positions for human lung cells^25,26,33,38–40^.

Wavenumber [cm^−1^]	Tentative assignments of Raman bands
750	Cytochrome c, heme group vibration
785	DNA
829	Tyrosine
855	Proline, tyrosine
1004	Phenylalanine
1126	Cytochrome c, heme group vibration
1260	Amide III (protein band), CH_2_ in-plane deformation (lipids)
1310	CH_2_ deformation (lipid), adenine, cytosine, cytochrome c
1444	Fatty acids, triglycerides, C–H bending vibrations
1583	δ(C=C), phenylalanine, cytochrome c, heme group vibration
1656	Amide I amide I of proteins, lipids, C=C vibrations
2854	Fatty acids, triglycerides, C–H_2_ symmetric stretching
2874	CH_2_ asymmetric stretch and CH stretch of lipids and proteins
2930–2940	CH_2_ asymmetric stretching of proteins, CH_3_ symmetric stretching of proteins

**Figure 2:**
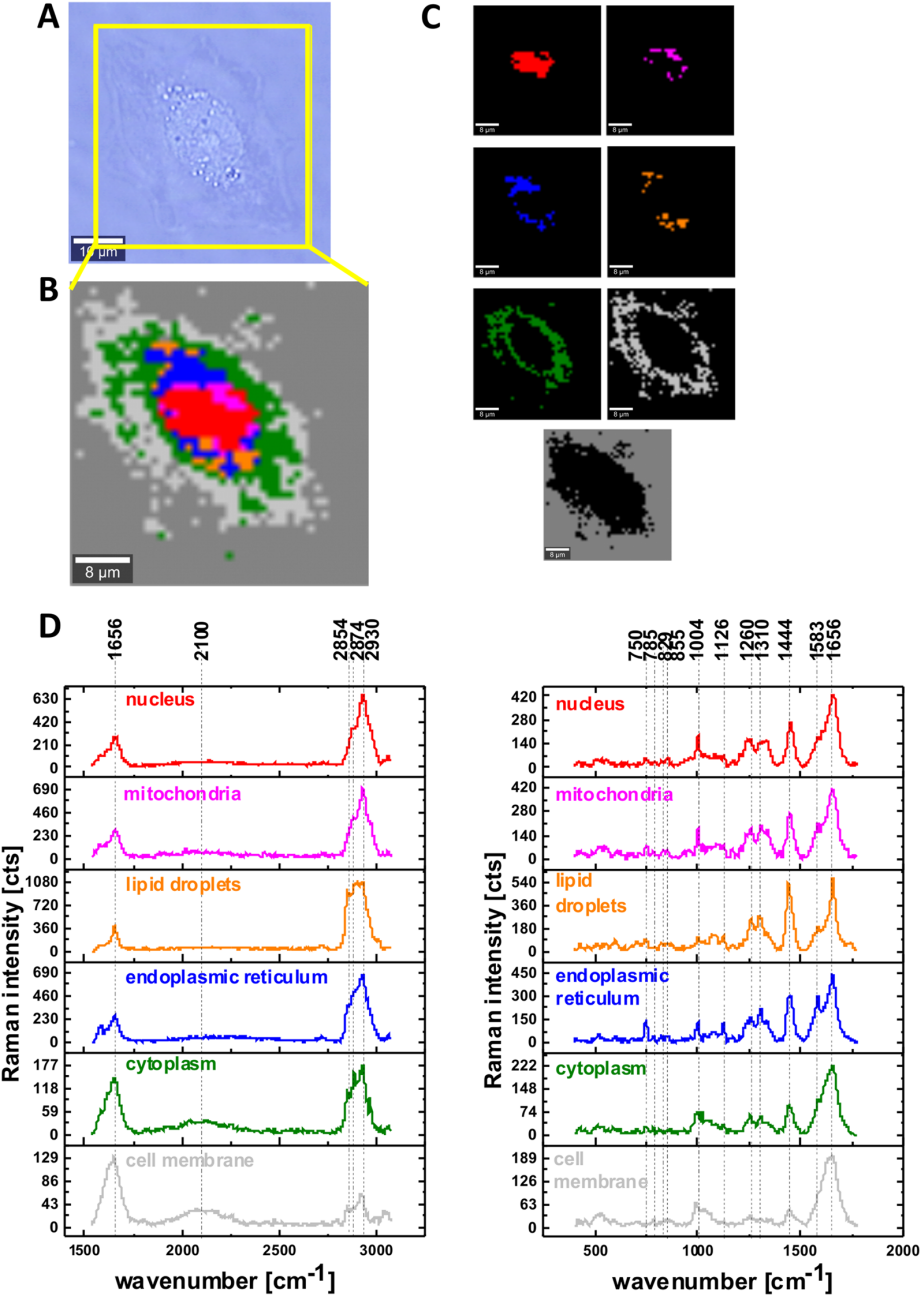
The microscopy image (**A**), Raman image for the area marked by yellow frame in the panel A, the size of Raman image (44 μm × 45 μm), resolution 1 μm of a typical human lung cell CCL-185 in hyperglycemia conditions supplemented with glucose 100 mM (**B**), Raman images of separate clusters identified by Cluster Analysis method assigned to: nucleus (red), mitochondria (magenta), endoplasmic reticulum (blue) and lipid droplets (orange), cytoplasm (green), cell membrane (light grey) and cell environment (dark grey) (**C**), the average Raman spectra for all clusters for high and for low frequency region (**D**) colors of the spectra correspond to the colors of clusters; integration time 0.3 s in the high frequency region and 0.5 s in the fingerprint region, laser power 10mW.

The results of Raman imaging for a typical cell of CCL-185 in hyperglycemia conditions (100 mM) are presented in Fig. 2.

It is worth emphasizing that for the hyperglycemia conditions (100 mM) the intensities of cytochrome c bands (750, 1126, 1310, 1583 cm^−1^) presented in Fig. 2 increase in mitochondria and rough endoplasmic reticulum when compared with the Raman intensities in Fig. SM1 without any glucose supplementation (SM1, Supplementary Material).

**Figure 3:**
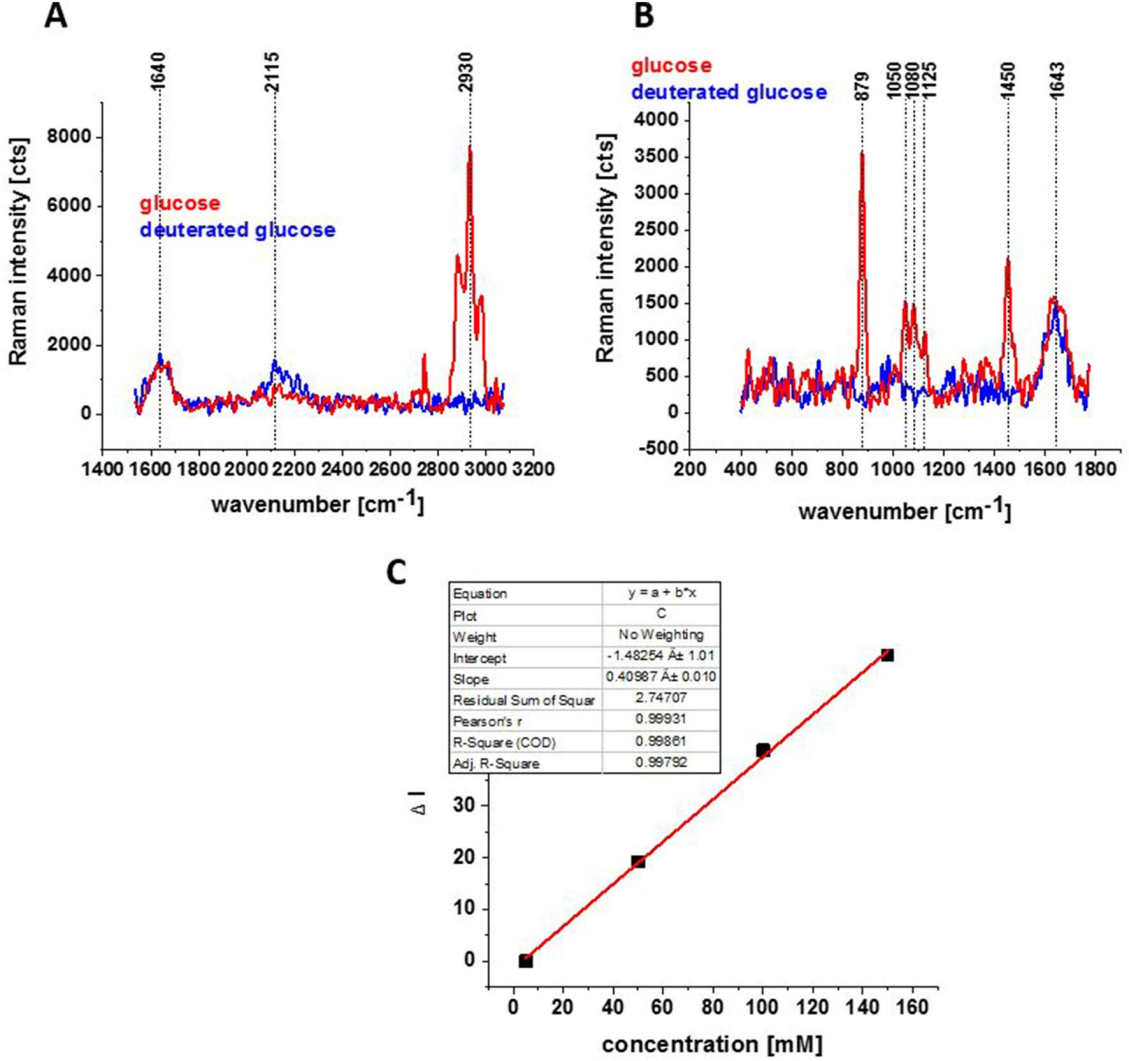
Raman spectra for glucose and deuterated glucose d-_7_ (150 mM) in PBS in high frequency region (**A**) and in low frequency region (**B**); integration time 20 s, 1 accumulation, laser power 10 mW; Linear dependence (of Raman intensity of the band at 2120 cm^−1^-background) on glucose d-7 concentration; integration time 1 s, 10 accumulation, laser power 10 mW (**C**), ΔI = I − I_0_, where I_0_ is the intensity of the background.

In the next step, Raman imaging was used to quantify intracellular deuterium content originating from deuterated carbon sources. Deuterium and hydrogen are chemically identical, as deuteration has a negligible effect on atom size, molecular shape, equilibrium bond length, or stiffness^41^ apart from vibrational frequencies presented in Fig. 3.

Figure 3 presents Raman spectra for glucose and deuterated glucose in PBS.

Comparison of the Raman spectra for deuterated and nondeuterated glucose shows several important differences, which will be used in our studies. First, the peaks at around 2930 cm^−1^ are very intense in the Raman spectrum for glucose (red line). They correspond to C–H stretching vibrations. Each D substitution in C–H bonds produces to the vibrational bands in the C–D stretch region which are shifted to ∼ 2000−2300 cm^−1^ in the Raman spectrum of deuterated glucose d_-7_ as predicted by the √2 rule arising from the reduced mass effects in the harmonic oscillator model. Indeed, one can see that Raman spectrum for deuterated glucose d_-7_ (blue line) has a characteristic wide peak at 2115 cm^−1^. In the fingerprint region one can see strong peaks at 879 cm^−1^ in Raman spectrum for glucose. Fourth, in Raman spectrum for glucose the peak at 1450 cm^−1^ is clearly visible.

As we mentioned above the main aim of our paper was to investigate the changes in cell metabolism upon supplementation with glucose at normal and hyperglycemia conditions by using isotope substitution. We analysed Raman images with isotope labeled glucose at hyperglycemia (100 mM) and normal (5 mM) conditions to trace the metabolism of glucose with high spatial resolution in specific organelles of the cell.

**Figure 4:**
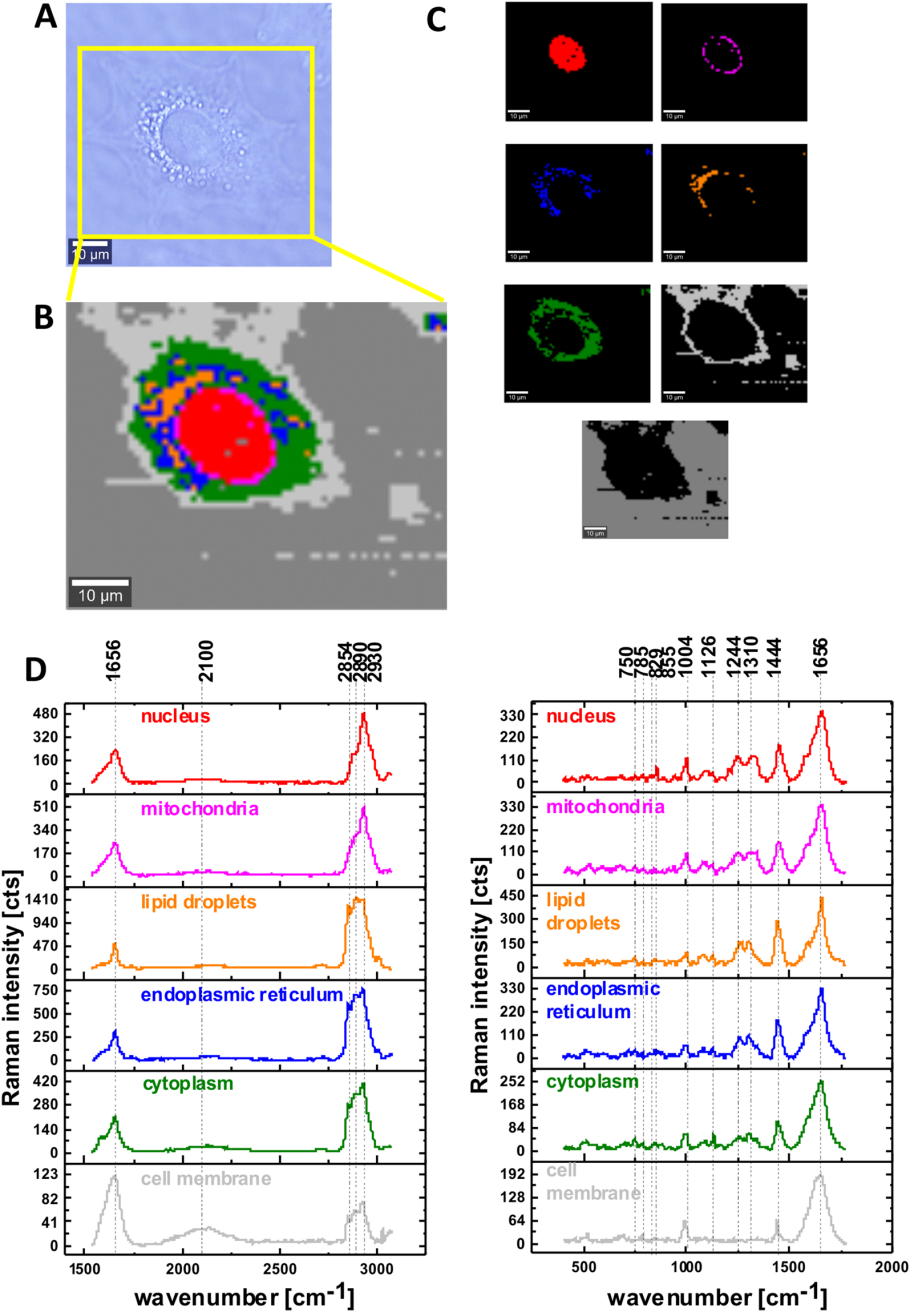
The microscopy image (**A**), Raman image for the area marked by yellow frame in the panel A, the size of Raman image (67 μm × 54 μm), resolution 1 μm of a typical human lung cell CCL-185 supplemented with deuterated glucose 100 mM (**B**), Raman images of separate clusters identified by Cluster Analysis method assigned to: nucleus (red), mitochondria (magenta), endoplasmic reticulum (blue) and lipid droplets (orange), cytoplasm (green), cell membrane (light grey) and cell environment (dark grey) (**C**), the average Raman spectra for all clusters for high and for low frequency region (**D**) colors of the spectra correspond to the colors of clusters; integration time 0.3 s in the high frequency region and 0.5 s in the fingerprint region, laser power 10mW.

Figure 4 presents a typical video image, Raman images and Raman spectra for a human lung cell CCL-185 with 100 mM deuterated glucose at hyperglycemia conditions. Typical Raman images and Raman spectra for human lung cells CCL-185 without glucose supplementation and for supplementation with deuterated glucose-d_7_ at normal glucose conditions (5 mM) are presented in Supplementary Materials (Figs. SM 1 and SM 2).

To learn more about changes in metabolism we compared the Raman spectra without glucose supplementation, supplemented cells with 5 mM glucose and glucose-d_7_ (normal conditions) as well as supplemented with 100 mM glucose and glucose-d_7_ (hyperglycemia conditions). The deuterated C–D bonds in glucose can be used as a universal indicator to probe carbon utilization in glucose metabolism. It is clear from Figs. 1, 2, 3, 4 that we do not detect glucose or glucose d-_7_ (lack of the characteristic bands of glucose in the fingerprint region) due to different catabolic pathways degrading glucose. Generally, any possible C–D vibrations of lipids, proteins, nucleic acids, and carbohydrates can give a contribution to this spectra region. As lipid biosynthesis contributes roughly 20–35% to the total cellular C–H content^4^, it plays a crucial part in the appearance of the broad C–D band through different lipid catabolic pathways degrading glucose.

**Figure 5:**
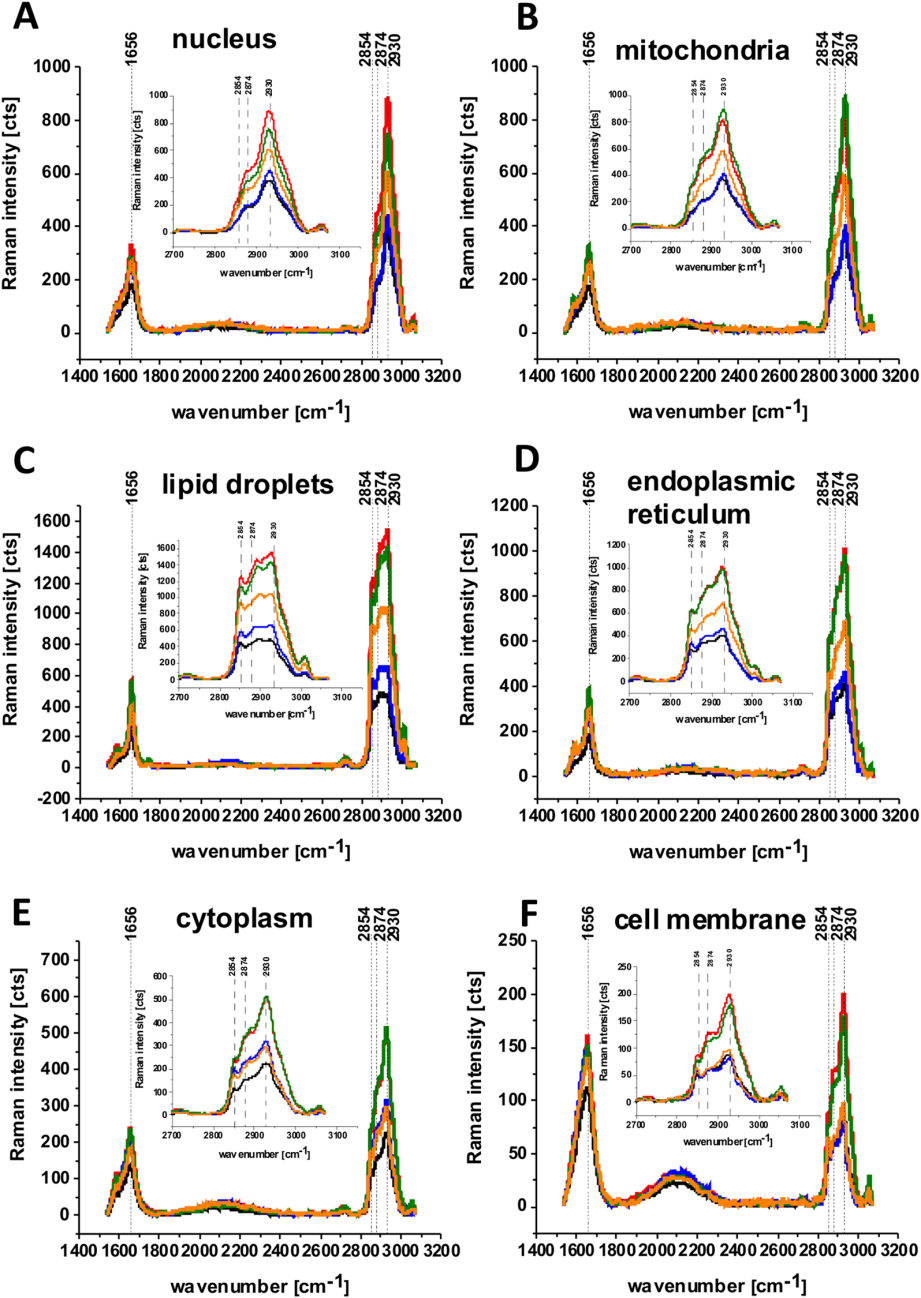
The average Raman spectra of human lung single cell CCL-185 without glucose supplementation (black line), supplemented with glucose (green line) and deuterated glucose (red line) at normal conditions (5 mM) and supplemented with glucose (orange line) and deuterated glucose (blue line) at hyperglycemia conditions (100 mM) for 1400–3200 cm^−1^ spectral region and for 2700–3150 cm^−1^ spectral region obtained from Raman images of separate clusters identified by Cluster Analysis method assigned to: nucleus (**A**), mitochondria (**B**), lipid droplets (**C**), endoplasmic reticulum (**D**), cytoplasm (**E**) and cell membrane (**F**); integration time 0.3 s in the high frequency region, laser power 10mW**.**

In order to better understand the changes caused in metabolism by glucose and deuterated glucose we used the results presented in Figs. 1, 2, 4 and SM 1, SM 2 to compare the average Raman spectra for nucleus, mitochondria, lipid droplets, endoplasmic reticulum, cytoplasm and cell membrane presented in Fig. 5.

Here, by coupling Raman imaging with isotope labeled glucose, we were able to trace the metabolism of glucose in separate organelles of living cells with high spatial resolution.

First, we compared the Raman signals in the region of C–H stretching vibrations of the CH_2_ or CH_3_ groups of proteins and lipids in the range of 2800–3000 cm^−1^ for supplementation with nondeuterated glucose at normal and hyperglycemia conditions at the same concentration (10%) of lipids from the diet (exogenous uptake in the cellular pool provided by FBS in the growing medium). One can see from Fig. 5C that for all organelles the Raman signals corresponding to lipids and proteins at 2800–3000 cm^−1^ are lower at hyperglycemia (100 mM of glucose) than for normal level of glucose (5 mM).

**Figure 6:**
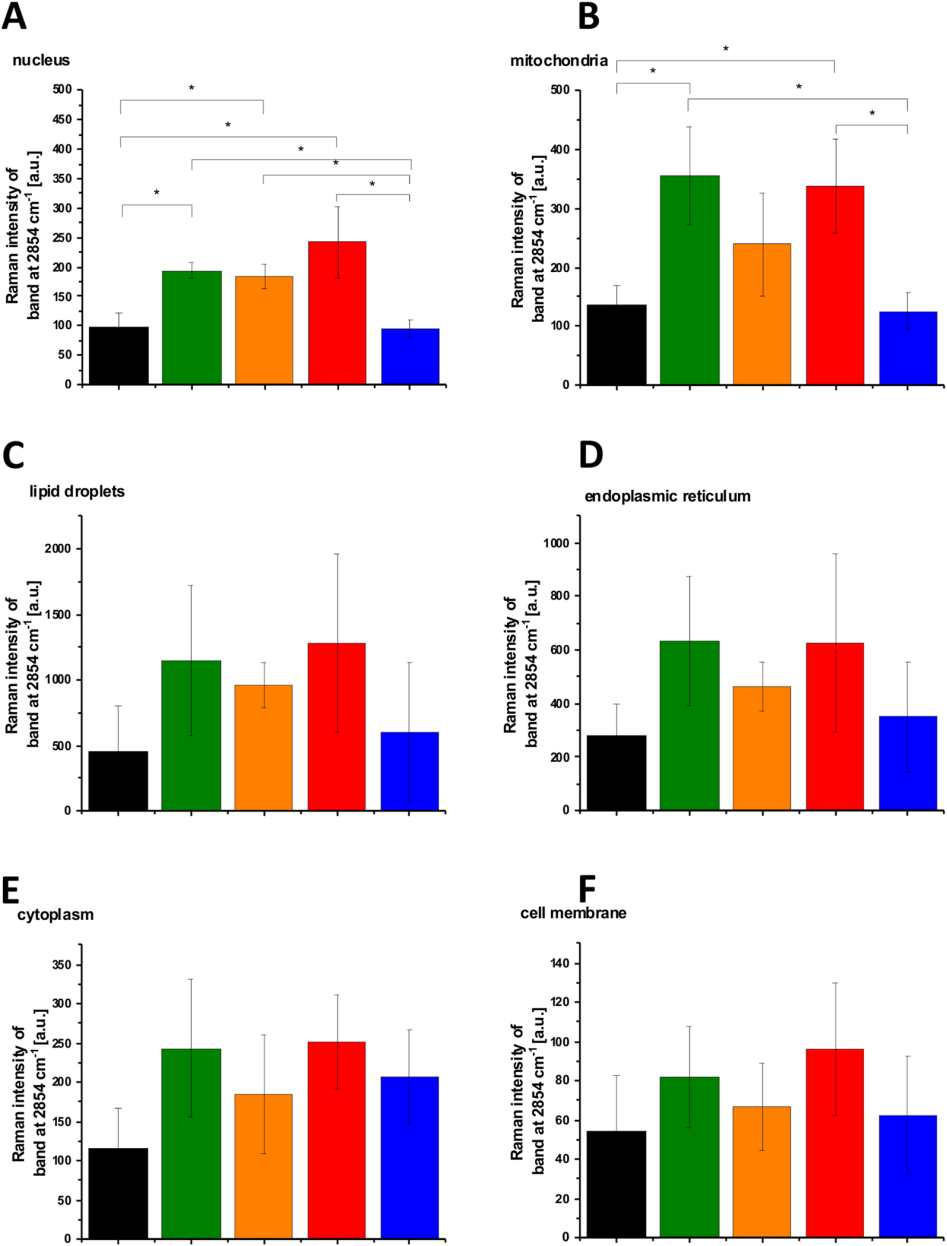
The Raman intensity band at 2854 cm^−1^ of human lung single cell CCL-185 without glucose supplementation (black line), supplemented with nondeuterated glucose (green line) and deuterated glucose (red line) at normal conditions (5 mM) and supplemented with nondeuterated glucose (orange line) and deuterated glucose (blue line) at hyperglycemia conditions (100 mM). The one-way ANOVA using the Tukey test was used to calculate the value significance, the asterisk * denotes that the differences are statistically significant, p-value ≤ 0.05.

**Figure 7:**
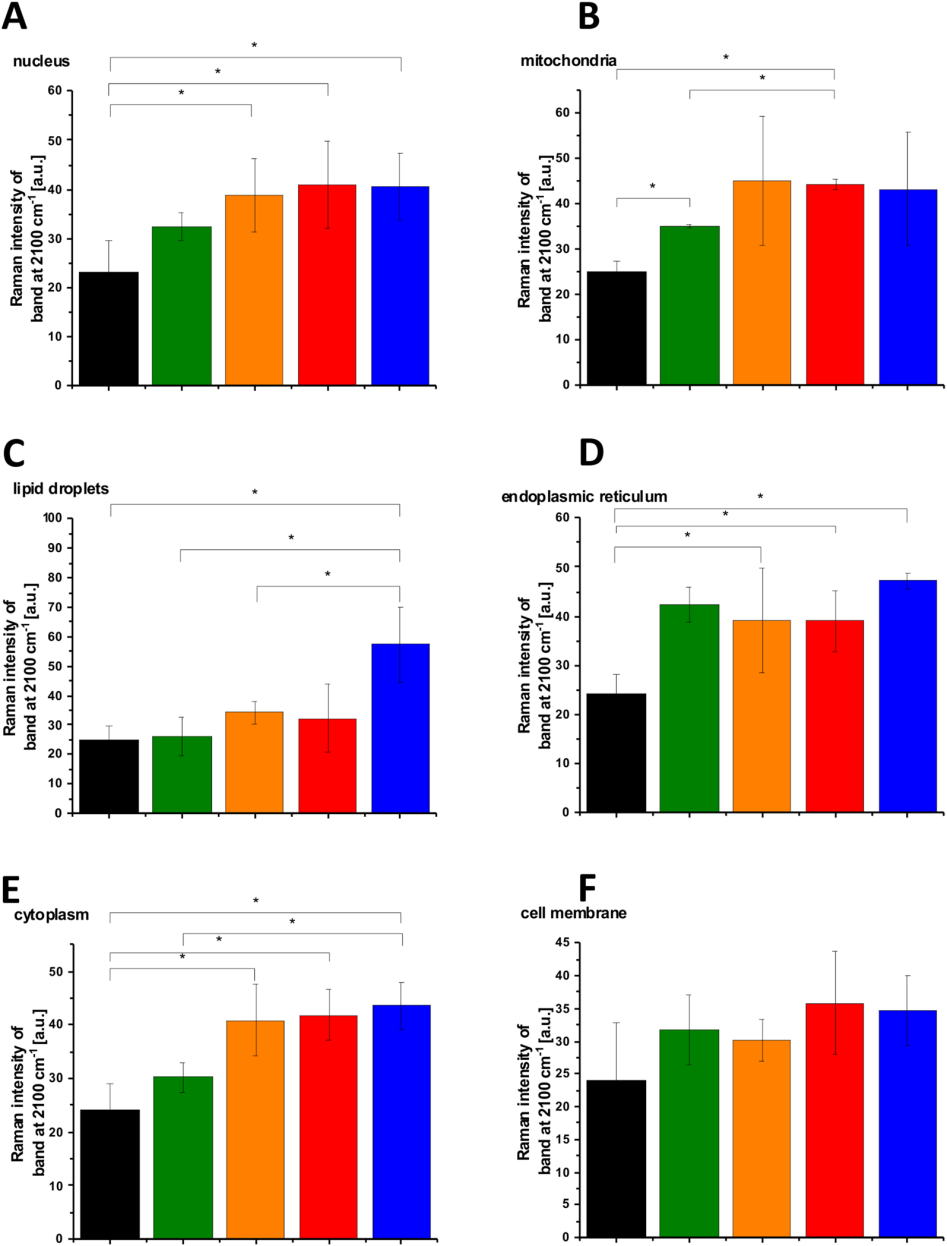
The Raman intensity band at 2100 cm^−1^ of human lung single cell CCL-185 without glucose supplementation (black line), supplemented with nondeuterated glucose (green line) and deuterated glucose (red line) at normal conditions (5 mM) and supplemented with nondeuterated glucose (orange line) and deuterated glucose (blue line) at hyperglycemia conditions (100 mM). The one-way ANOVA using the Tukey test was used to calculate the value significance, the asterisk * denotes that the differences are statistically significant, p-value ≤ 0.05.

Figure 6 shows the analysis of statistical significance for the organelles at 2854 cm^−1^. Figure 6C shows that the intensity of the band at 2854 cm^−1^ for lipid droplets corresponding to the C–H stretching vibration of lipids decreases at hyperglycemia conditions compared to normal glucose level. In contrast the Raman intensity of the band C–D at 2100 cm^−1^ presented in Fig. 7C increases for lipid droplets at hyperglycemia conditions compared to the normal glucose level. The results presented in Figs. 5, 6, 7 demonstrate that de novo lipid synthesis decreases at hyperglycemia conditions for cancer lung cells.

**Figure 8:**
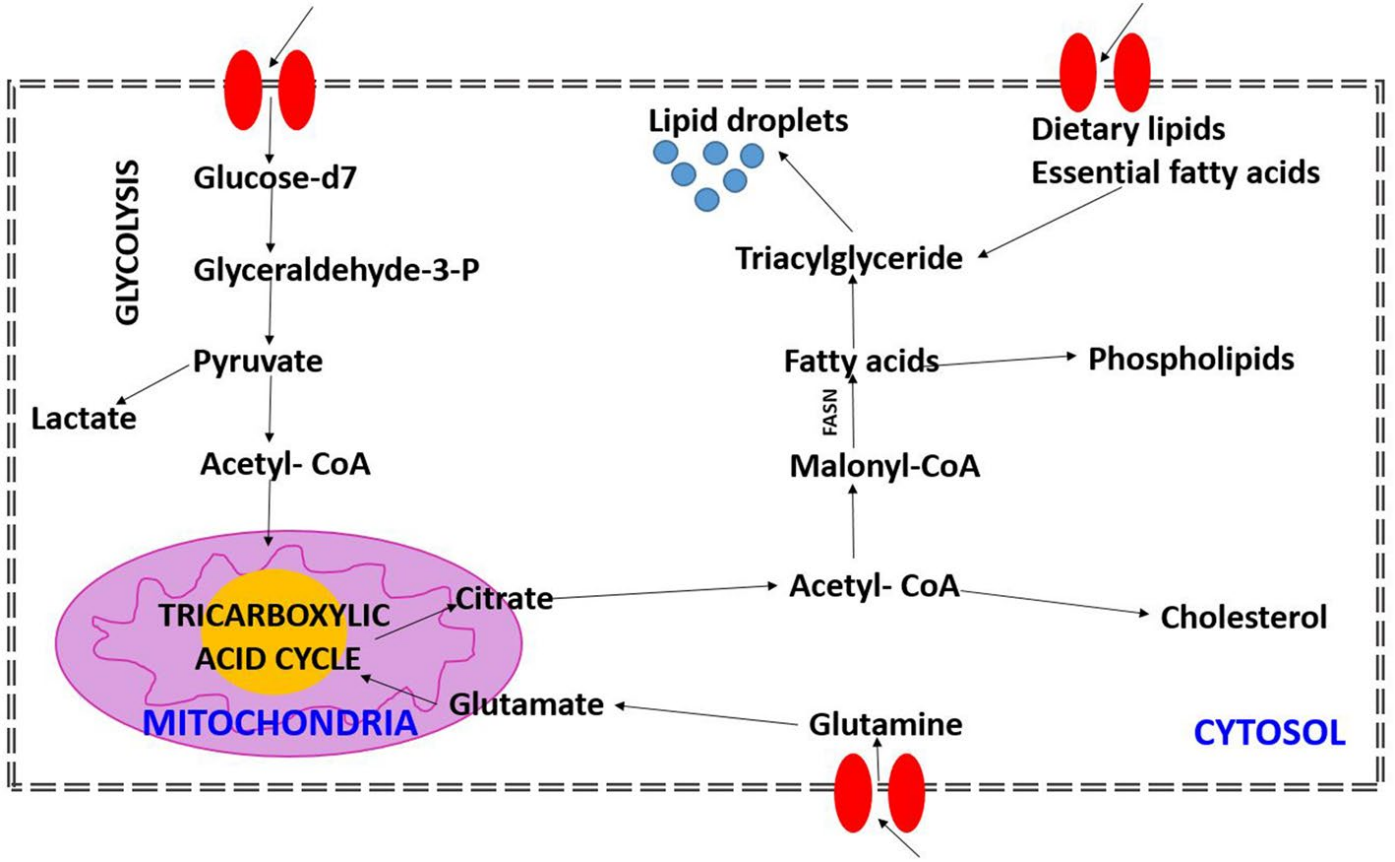
Glucose –derived de-novo lipid synthesis in mammalian cells.

It is well known that a common feature of cancer cells is their ability to reprogram their metabolism to sustain the production of ATP and macromolecules needed for cell growth, division and survival^34,42^. Glucose –derived de-novo lipid synthesis in mammalian cells is presented in Fig. 8.

Briefly, in normal cells glucose is processed by glycolysis to generate ATP and pyruvate. Then the ribose 5-phosphate and NADPH were produced through the pentose phosphate pathway (PPP), or enter into the tricarboxylic acid (TCA) cycle in mitochondrion. Glucose-derived citrate is converted to acetyl-CoA, oxaloacetate (OAA), or a-ketoglutarate (a-KG). Glutamine is deaminated to form glutamate, which is processed to produce a-KG for use in the TCA cycle.

In contrast, the main pathway of glucose metabolism in cancer cells is aerobic glycolysis, termed Warburg effect. In cancer cells, glucose uptake and the production of lactate were reported to dramatically increase even in the presence of oxygen and fully functioning mitochondria. This classic type of metabolic change provides a fast pathway to produce substrates required for cancer cell proliferation and division, which is involved in tumor growth, metastatic progression and long-term survival. It must be emphasized that both glycolysis and mitochondrial metabolism are crucial to cancer cells in the Warburg Effect^43^.

Our results provide a direct evidence that a high level of glucose decreases the metabolism via oxidative phosphorylation in mitochondria in cancer cells and shifts the metabolism to glycolysis via Wartburg effect. It is worth emphasizing that the Raman intensity of the C–H bands in the 2800–3000 cm^−1^ spectral range for the glucose free cells without glucose supplementation and cells supplemented with deuterated glucose at high hyperglycemia level (100 mM) are practically identical, which means that the mitochondrial metabolism is significantly reduced and the Raman signals in this region come mainly from the lipids uptake from diet provided by FBS.

Now, we will show that the comparison between the results for normal and deuterated glucose is an excellent method to monitor de novo lipid synthesis and to separate de novo synthesis pathways from exogenous uptake of lipids provided with the diet.

The isotope substitution by deuter was used in this paper to shed some light on this issue. The stretching vibrations of C–H bonds in the region of 2800–3000 cm^−1^ on the aliphatic backbone of lipids and proteins are found to be effectively decoupled from other molecular vibrations. Each D substitution in a CH_2_ or CH_3_ group of the backbone produces the removal of C–H bonds (and decrease of the Raman signal in the region of 2800–3000 cm^−1^) and an appearance of the band in the C–D stretching region (2100–2300 cm^−1^).

The results presented in Figs. 6 and 7 demonstrate that the most significant changes in the spectra regions of 2800–3000 cm^−1^ and 2100–2300 cm^−1^ upon D substitution are observed in lipid droplets (Fig. 5C). It indicates that glucose is largely utilized for de novo lipid synthesis.

**Figure 9:**
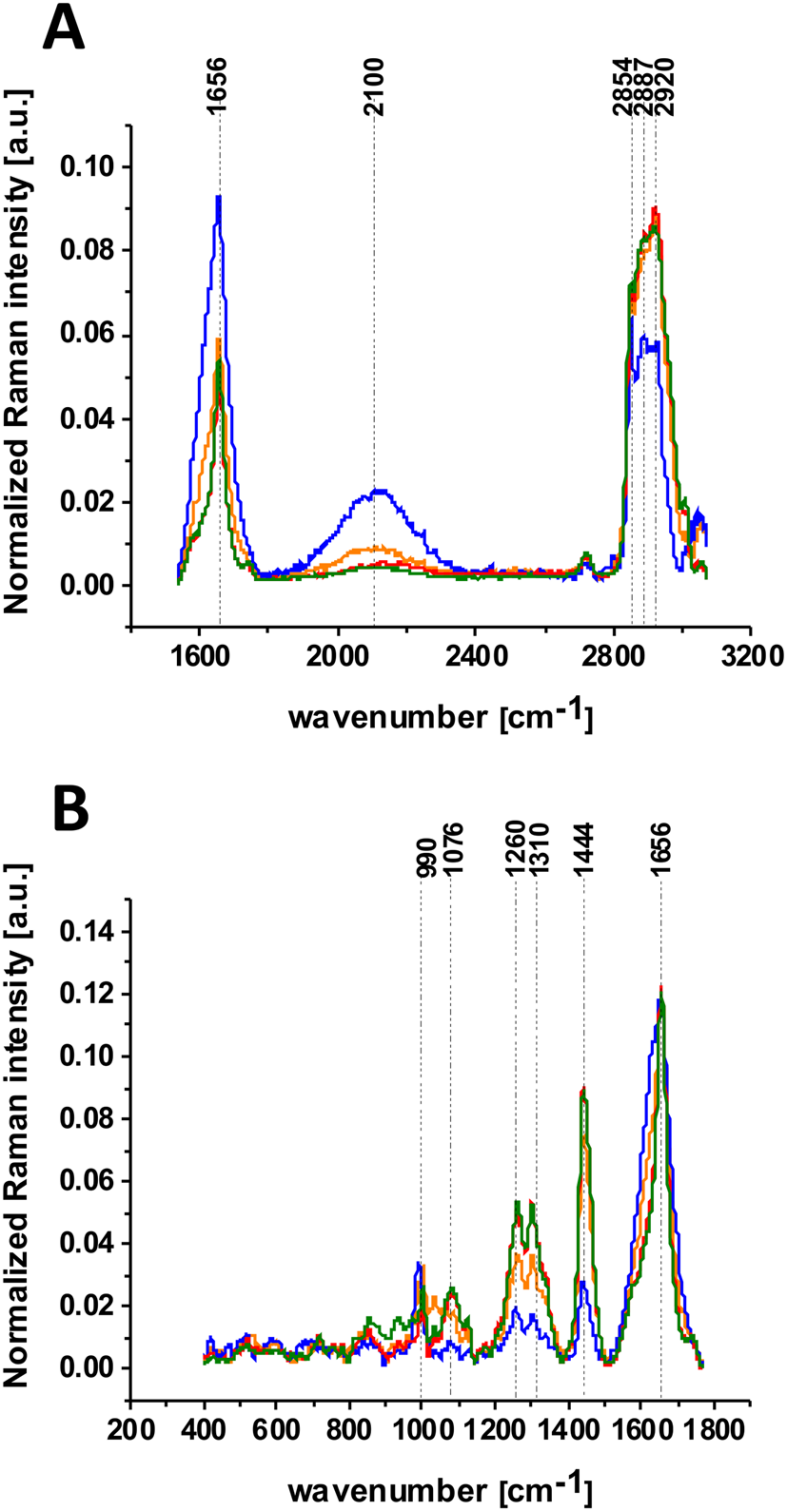
The average Raman spectra for human lung cells for lipid rich area supplemented with glucose 100 mM (orange line), deuterated glucose 100 mM (blue line), glucose 5 mM (green line) and deuterated glucose 5 mM (red line) in high frequency region (**A**) and in low frequency region (**B**); integration time 20 s, laser power 10 mW.

In order to better visualize the changes caused in glucose metabolism we concentrated on lipid droplets. We compared the average Raman spectra characteristic for these lipid structures. The average Raman spectra are based on 16 spectra recorded for the integration times of 20 s, for each type of supplementation (5 mM, 100 mM for nondeuterated glucose and deuterated glucose). For each type of supplementation we measured 4 cells to calculate the average Raman spectrum. The comparison of average normalized Raman spectra of lipid droplets incubated with glucose and deuterated glucose of 5 mM and 100 mM concentration in medium is presented in Fig. 9.

One can see from Fig. 9A that the prominent changes are visible in region of amide I and C=C lipid vibrations at 1656 cm^−1^, C–D stretching vibrations of lipids at 2100–2300 cm^−1^ and C–H stretching vibrations of lipids at 2800–3000 cm^−1^. One can see that the C–D band of lipid droplets at 2100 cm^−1^ gives the strongest Raman signal in cells incubated with 100 mM deuterated glucose while the intensities of bands corresponding to C–H at 2800–3000 cm^−1^ decrease due to isotope substitution of C–H bonds by C–D bonds. The significant changes are observed also at 1444 cm^−1^ corresponding to C–H deformation vibrations of lipids presented in Fig. 9B. The Raman signal at 1444 cm^−1^ decreases upon deuteration. The results provide direct evidence that glucose is largely utilized for de novo lipid synthesis. The intensity of Raman bands at 1444 cm^−1^ corresponding to C–H bending vibrations of lipids in Fig. 9 shows that the signals are lower at hyperglycemia (100 mM of glucose) than for normal level of glucose (5 mM). The results from Fig. 9 support our results obtained from the high frequency region 2800–3000 presented in Fig. 5. It indicates that de novo lipid synthesis decreases at hyperglycemia conditions for cancer lung cells.

**Figure 10:**
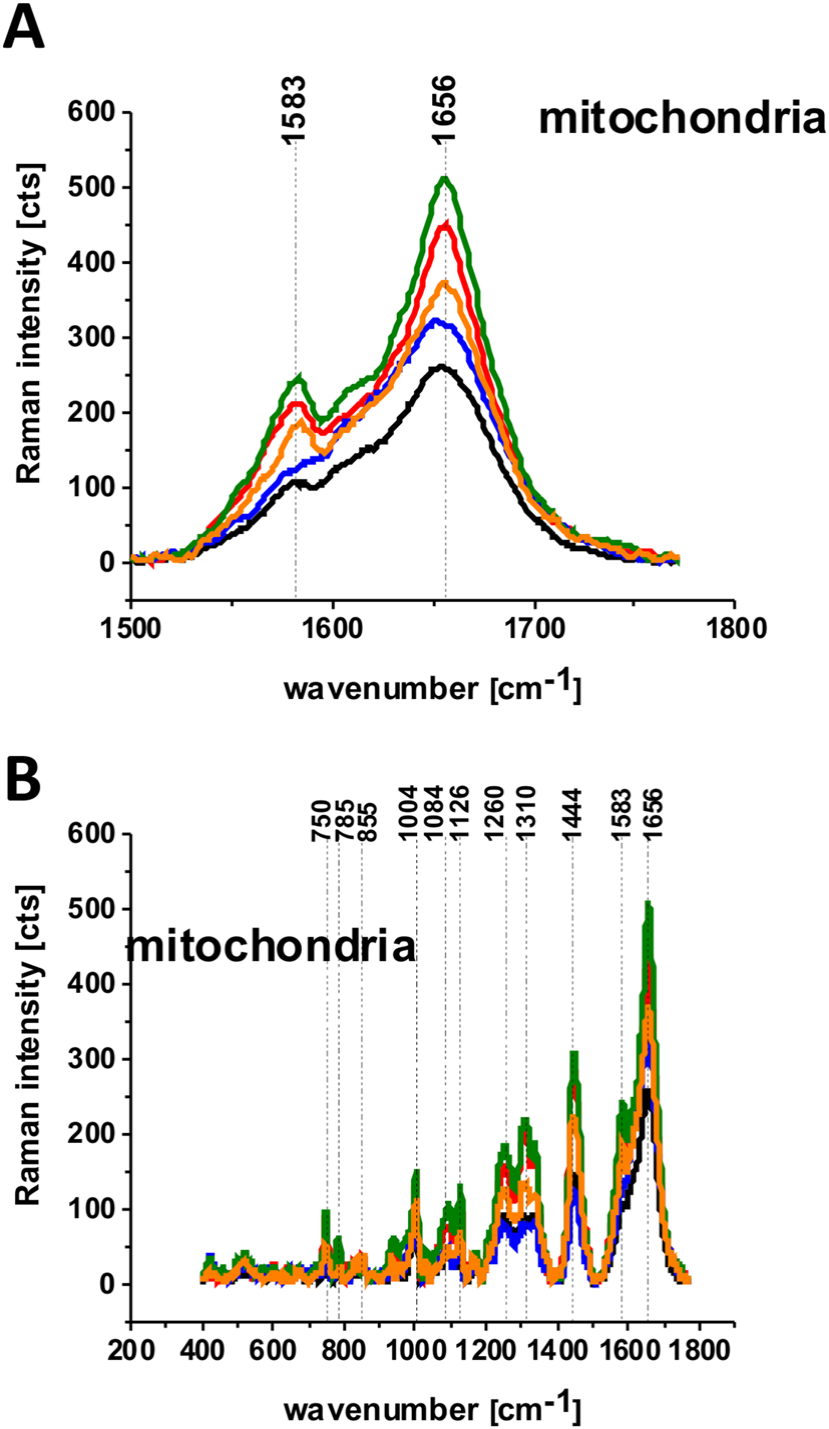
The average Raman spectra of human lung single cell CCL-185 without glucose supplementation (black line), supplemented with glucose (green line) and deuterated glucose (red line) at normal conditions (5 mM) and supplemented with glucose (orange line) and deuterated glucose (blue line) at hyperglycemia conditions (100 mM) for 1500–1800 cm^−1^ spectral region (**A**) and for 200–1900 cm^−1^ spectral region (**B**) obtained from Raman images of separate clusters identified by Cluster Analysis method assigned to: mitochondria.

The isotope substitution provides information not only on lipid metabolism, but also on another metabolic processes. We could detect other metabolites of glucose by using glucose as precursor for other macromolecular synthesis, such as nucleotides and proteins. Hyperspectral imaging in the fingerprint region would allow us to separate different metabolic species based on the spectra differences. The results from the fingerprint region presented in Fig. 10 reflect redox mitochondrial metabolic processes.

One can see that for normal physiological conditions of glucose level (5 mM) both for nondeuterated and deuterated glucose the intensity of the band at 1583 cm^−1^ Raman signal intensity is higher than for the hyperglycemia (100 mM).

The band at 1583 cm^−1^ in Fig. 10 represents the vibration of heme group of cytochrome c and can be used as the “redox state Raman marker”. Recently we demonstrated that this Raman vibration can serve as a sensitive indicator of the oxidized and reduced forms of cytochrome c^26^. It indicates that the Raman peak at 1583 cm^−1^ can be used as a marker to explore apoptosis and oxidative phosphorylation in mitochondria. This band reflects the dual face of cytochrome c in life and death processes: apoptosis and oxidative phosphorylation. The balance between cancer cells proliferation (oxidative phosphorylation) and death (apoptosis) decides about the rate of cancer development^26,44^. The Raman signal of the cancer lung cell at 1583 cm^−1^ depends on cytochrome c concentration (which also depends on the number of mitochondria in a cell), and the redox state (oxidized or reduced forms). The Raman intensity of the oxidized form is much smaller than that of the reduced form^25,26,35,45^. Inside a normal mitochondrium cytochrome c exists in the oxidized form. Dysfunction of mitochondrium associated with several malignancies, including cancer or hyperglycemia blocks the transfer of electrons between complexes III and IV of the respiratory chain, resulting in decreasing the efficiency of the oxidative phosphorylation (respiration) process and lower ATP synthesis^35,45^. It has been suggested that under a hyperglycemic condition, more than usual glucose enters the metabolic cycle. As a result of more intense changes in the tricarboxylic acid cycle, too much NADH and FADH_2_ are supplied to the mitochondrial chain. As a consequence, the voltage gradient across the mitochondrial membrane increases to the critical point where electron transport in complex III is blocked, causing the electrons to be withdrawn back into coenzyme Q where they are attached to oxygen molecules. In this way, superoxide anion radicals are generated^46^.

## Conclusions

This paper presents a truly unique landscape of cancer cell biochemistry by non-invasive Raman imaging combined with isotope substitution cells. We studied glucose metabolism in lung cancer cells at normal and hyperglycemia conditions. We found that isotope substitution of glucose by deuterated glucose allows to separate de novo lipid synthesis from exogenous uptake of lipids obtained from the diet. We demonstrated that glucose is largely utilized for de novo lipid synthesis. The mitochondrial metabolism was monitored by the Raman signal at 1583 cm^−1^ representing the concentration of cytochrome c. Our results provide a direct evidence that high level of glucose at hyperglycemia conditions decreases mitochondrial metabolism via oxidative phosphorylation in cancer cells. It suggests that hyperglycemia is a factor that may contribute to a more malignant phenotype of cancer cells by inhibition of oxidative phosphorylation and apoptosis.

## Supplementary Information

Supplementary Figures.

## Data Availability

The raw data underlying the results presented in the study are available from Lodz University of Technology Institutional Data Access for researchers who meet the criteria for access to confidential data. The data contain potentially sensitive information. Request for access to those data should be addressed to the Head of Laboratory of Laser Molecular Spectroscopy, Institute of Applied Radiation Chemistry, Lodz University of Technology. Data requests might be sent by email to the secretary of the Institute of Applied Radiation Chemistry: mitr@mitr.p.lodz.pl.
