# Supplementary material for: Hyperglycemia and cancer in human lung carcinoma by means of Raman spectroscopy and imaging
Source: Sci Rep. 2022 Nov 3;12:18561. doi: 10.1038/s41598-022-21483-y (PMC9633797; doi:10.1038/s41598-022-21483-y)
Supplement: Supplementary file 1 — Supplementary Figures. [file 41598_2022_21483_MOESM1_ESM.docx]

*
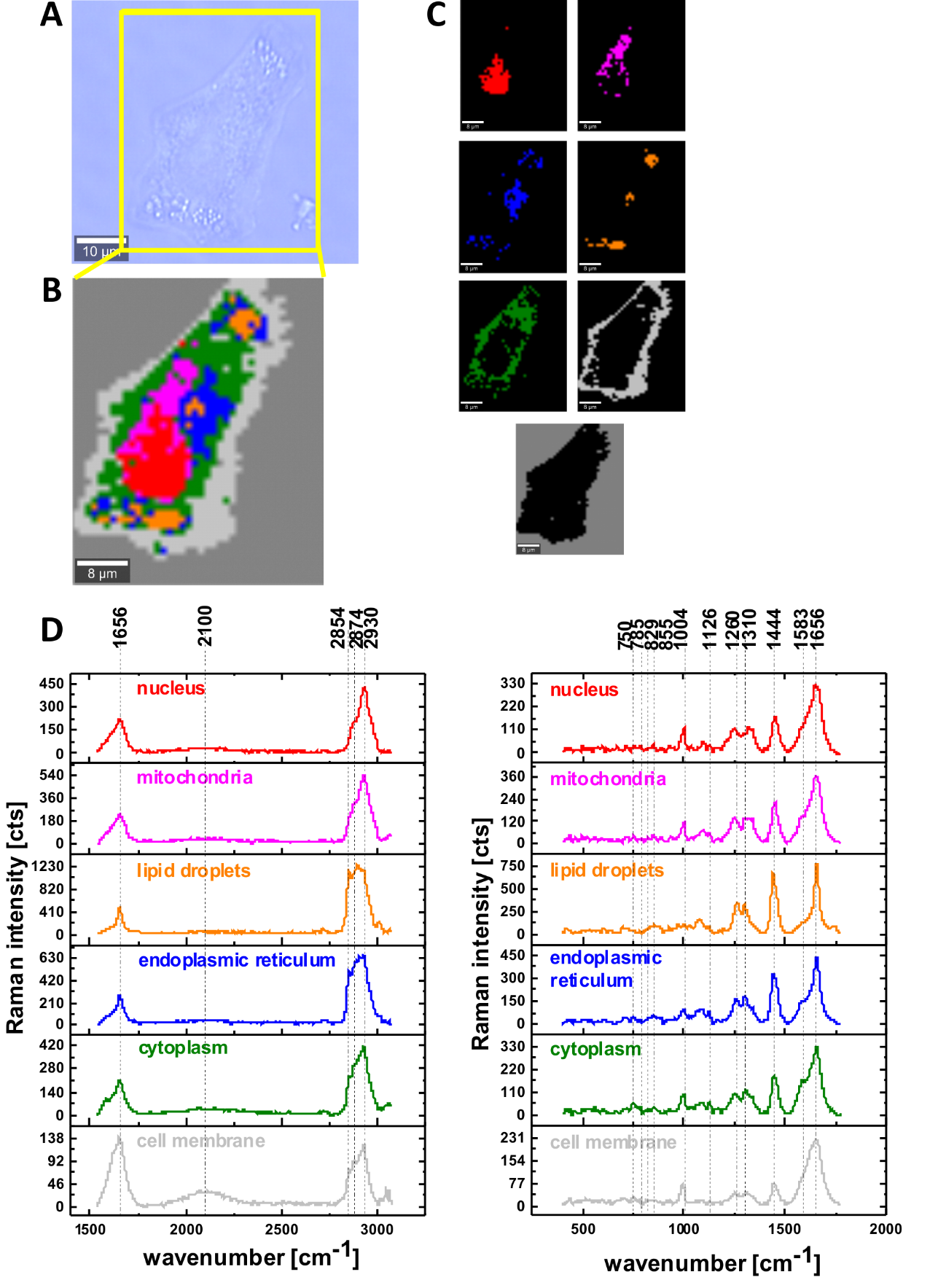
*

*Fig SM 1 The microscopy image (A), Raman image for the area marked by yellow frame in the panel A, the size of Raman image (45 μm × 42 μm), resolution 1 μm of a typical human lung single cell CCL-185 (B), Raman images of separate clusters identified by Cluster Analysis method assigned to: nucleus (red), mitochondria (magenta), endoplasmic reticulum (blue) and lipid droplets (orange), cytoplasm (green), cell membrane (light grey) and cell environment (dark grey) (C) the average Raman spectra for all clusters for high and for low frequency region (D), colors of the spectra correspond to the colors of clusters; integration time 0.3 sec in the high frequency region and 0.5 sec in the fingerprint region, laser power 10mW.*

*
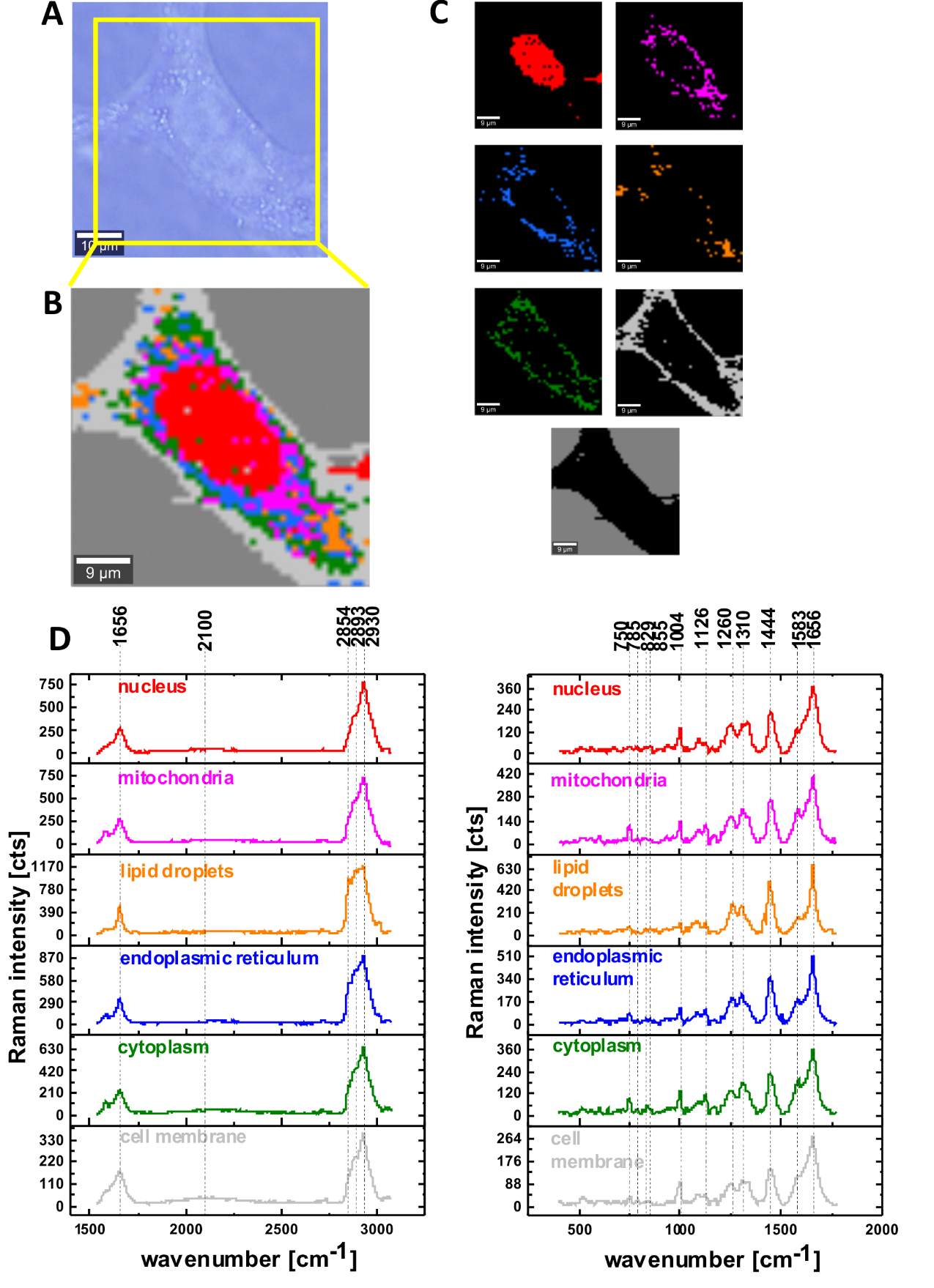
*

*Fig SM 2 The microscopy image (A), Raman image for the area marked by yellow frame in the panel A, the size of Raman image (50 μm × 50 μm), resolution 1 μm of a typical human lung single cell CCL-185 supplemented with deuterated glucose 5 mM (B), Raman images of separate clusters identified by Cluster Analysis method assigned to: nucleus (red), mitochondria (magenta), endoplasmic reticulum (blue) and lipid droplets (orange), cytoplasm (green), cell membrane (light grey) and cell environment (dark grey) (C) the average Raman spectra for all clusters for high and for low frequency region (D), colors of the spectra correspond to the colors of clusters; integration time 0.3 sec in the high frequency region and 0.5 sec in the fingerprint region, laser power 10mW.*
